# Flow-Mediated Vasodilation Is Not Attenuated in Hypertensive Pregnancies Despite Biochemical Signs of Inflammation

**DOI:** 10.5402/2012/709464

**Published:** 2012-01-17

**Authors:** Heli Saarelainen, Henna Kärkkäinen, Pirjo Valtonen, Kari Punnonen, Tomi Laitinen, Nonna Heiskanen, Tiina Lyyra-Laitinen, Esko Vanninen, Seppo Heinonen

**Affiliations:** ^1^Department of Ob/Gyn, Kuopio University Hospital, University of Eastern Finland, 70211 Kuopio, Finland; ^2^Laboratory Centre, Kuopio University Hospital, University of Eastern Finland, 70211 Kuopio, Finland; ^3^Department of Clinical Physiology and Nuclear Medicine, Kuopio University Hospital, University of Eastern Finland, 70211 Kuopio, Finland

## Abstract

*Background*. Our objective was to evaluate endothelial function and markers of inflammation during and after pregnancy in normal pregnancies compared to pregnancies complicated with hypertension or preeclampsia (PE). *Methods and Results*. We measured endothelium-dependent brachial artery flow-mediated vasodilation (FMD) and high sensitive C-reactive protein (hsCRP), interleukin-6 (IL-6), and tumour necrosis factor-*α* (TNF-*α*) in 32 women with normal pregnancy and in 28 women whose pregnancy was complicated with hypertensive disorder in the second half of pregnancy and minimum 3-month postpartum. Enhancement of endothelial function was greater in hypertensive than normal pregnancies, the mean FMD% being 11.0% versus 8.8% during pregnancy (*P* = 0.194) and 8.0% versus 7.9% postpartum (*P* = 0.978). Concentrations of markers of inflammation were markedly increased in pregnant hypertensive group compared to those after delivery (hsCRP 4.5 versus 0.80 mg/L, *P* = 0.023, IL-6 2.1 versus 1.2 pg/mL, *P* = 0.006; TNF-*α* 1.9 versus 1.5 pg/mL, *P* = 0.030). There were no statistically significant associations between the markers of inflammation and FMD. *Conclusions*. Brachial artery FMD was not attenuated in the third trimester hypertensive pregnancies compared to normal pregnancies, whereas circulating concentrations of hsCRP and IL-6 and TNF-*α* reacted to hypertensive complications.

## 1. Introduction

Assessment of flow-mediated dilatation (FMD) in cardiovascular disease gives information on endothelial function and predicts cardiovascular morbidity and even cardiovascular death [1]. During normal pregnancy endothelial function improves due to increase in nitric oxide (NO) bioavailability [2–4]. Healthy endothelium not only mediates endothelium-dependent vasodilation, but also actively suppresses thrombosis, vascular inflammation, and hypertrophy [5]. In preeclampsia-impaired trophoblast invasion and differentiation lead to placental ischemia and increased release of antiangiogenic factors [6]. Endothelial dysfunction, in turn, leads to mild to severe hypertension, oedema and proteinuria, and ischemic changes in several organs, in particular in kidneys [6]. Despite many advances in our understanding of the pathophysiology of preeclampsia, delivery of the placenta remains the only definitive treatment. According to WHO more than 40 000 women die of pregnancy-induced hypertensive disorder per year [7]. Although signs and symptoms of preeclampsia, with the exception of hypertension in a small number of women, recover the risk of premature cardiovascular diseases is 2–7-fold higher after maternal placental syndrome than after a normal pregnancy [8].

Endothelial function has been assessed mostly by FMD, based on the assumption that impaired FMD reflects not only impaired dilatation but also other alterations of endothelial function [5]. In preeclampsia attenuated FMD has been assumed to predict the clinical disease about 10 weeks before the actual signs and symptoms [9]. We wanted to evaluate whether the assessment of FMD is clinically relevant in hypertensive pregnancies and preeclampsia and how recovery takes place after delivery.

## 2. Materials and Methods

We prospectively examined a total of 60 women: 32 normal pregnant women and 12 preeclamptic and 16 hypertensive women in the third trimester of pregnancy and minimum three-month postpartum. Postpartum, we could reach 75% of the normal pregnancies and 61.5% of the hypertensive group. Smokers were excluded. The patients were recruited in the Kuopio University Hospital Maternity clinic or maternity ward through years 2002–2006. The local Ethics Committee approved the study and the patients gave written informed consent.

Weight in the beginning of pregnancy and height were collected from medical records and BMI was calculated. Blood pressure was measured at nurse's office from right arm after 15 minutes of rest with an automatic device. The mean of the three measurements was calculated for each patient. Continuous blood pressure recording was performed from the middle finger of the right hand. All recordings and data analyses were performed with an IBM PC-compatible microcomputer with a commercial WINACQ/WINCPRS acquisition electronics and software (Absolute Aliens Oy, Turku, Finland), which includes a sophisticated analysis program designed for research of cardiovascular physiology.

Body composition was determined by bioimpedance analysis (InBody 3.0, BioSpace Co., LTD., Seoul, Republic of Korea). The device used 8-point tactile electrodes; two were in contact with the palm and thumb of each hand and two with anterior and posterior aspects of the soles of the feet. The measurement was performed for undressed subjects in upright position, the soles in contact with the foot electrodes and the hands grabbing the hand electrodes. The electrodes were connected to the voltage and power supply of the device. The device used segmental multifrequency measurement method. The total body was measured in five segments, upper and lower extremities and trunk, and microprocessor controlled coupling of the electrodes. The multifrequency measurement was conducted by using multiple frequencies at 5, 50, 250, and 500 kHz for each electrode coupling. From the device report, intracellular and extracellular fluid volume, fat mass, percentage body fat (fat-%), and lean body mass were used. The measurements were performed during the third trimester of pregnancy and three-month after delivery at least two hours after ingestion of a light breakfast and after voiding the urinary bladder before the measurement.

Fasting blood samples were drawn from each subject during pregnancy and three months postpartum. Venous blood samples were drawn from the right antecubital vein of recumbent subjects after a 12-hour overnight fast for determination of serum hsCRP, TNF-*α*, and IL-6, and serum lipids and lipoprotein concentrations. Serum was separated by centrifugation and samples were stored frozen (−70°C) until analysis. Cholesterol and triglyceride levels were assayed by standard enzymatic photometric methods with Konelab 60i Clinical Chemistry Analyzer (Thermo Electron Co., Finland). Serum samples were analyzed by Immage automated analyzer using Beckman Coulter High Sensitivity C-Reactive Protein (CRPH) reagents (Beckman-Coulter, Fullerton, CA, USA) with working range of 0.2 to 1440 mg/L. Serum samples were analyzed according to the instructions of the manufacturer.

IL-6 was analyzed by R&D Systems Quantikine HS Human IL-6 Immunossay Kit (Minneapolis, USA) with working range of 0.156 to 10 pg/mL. Serum samples were analyzed according to the instructions of the manufacturer. Calibrators (supplied by the manufacturer) were analyzed in duplicate and samples as a single measurements. The absorbance at 490 nm was measured using a microplate reader (Tecan SPECTRAFluor, Tecan Group Ltd., Maennedorf, Switzerland). Serum samples were analyzed by R&D Systems Quantikine HS Human TNF-*α*/TNFSF1A Immunossay Kit (Minneapolis, USA) with working range of 0.5 to 32 pg/mL. Serum samples were analyzed according to the instructions of the manufacturer. Calibrators (supplied by the manufacturer) were analyzed in duplicate and samples as a single measurements. The absorbance at 490 nm was measured using a microplate reader (Tecan SPECTRAFluor, Tecan Group Ltd., Maennedorf, Switzerland).

Ultrasound studies were performed using either Sonoace 6000C (Kretz, Marl, Germany) or Sequoia 512 (Acuson, Mountain View, CA, USA) ultrasound scanner. To assess brachial FMD, the left brachial artery diameter was measured both at rest and after reactive hyperaemia. Increased flow was induced by inflation of a pneumatic tourniquet placed around the forearm to a pressure of 250 mm Hg for 4.5 minutes, followed by release. Three measurements of arterial diameter were performed at end diastole at a fixed distance from an anatomic marker at rest and at 40, 60, and 80 seconds after cuff release. The vessel diameter in scans after reactive hyperaemia was expressed both as change in absolute diameter (FMD) and as the percentage relative to the resting scan (FMD%). After a 10 min wash-out period nitrate (isosorbide dinitrate spray 2.5 mg, Dinit) was administered per os. Nitrate related vasodilatation (NTG%) was assessed from the maximally dilated vessel diameter 3–5 min after nitrate administration relative to vessel diameter at rest before nitrate administration.

Data are presented as means and medians when appropriate. Data normality was examined with Kolmogorov-Smirnov test. Comparisons between the groups were performed with the Kruskall-Wallis test and the mixed models-analysis with Bonferroni adjustment for multiple comparisons. Univariate correlations were performed using Spearman correlation. A computer software program (SPSS 11.5 for windows; SPSS Inc, Chicago, IL, USA) was used. A probability level < 0.05 was considered statistically significant.

## 3. Results

Clinical characteristics of the mothers and outcome of the neonates are presented in Table 1. The gestational age and birth weight of the neonates were significantly lower in the hypertensive and preeclampsia group than in the reference group, whereas the caesarean section rate was nonsignificantly increased. The mean age of the mothers, gestational age, and BMI before pregnancy were similar in both groups. Smokers were excluded. Blood pressure was significantly elevated in hypertensive group.

Data of the brachial artery flow-mediated vasodilation are shown in Tables 2(a)–2(c). Endothelial function was somewhat improved during pregnancy compared to non-pregnant state, but the change according to improvement of FMD% was not statistically significant. There was no significant difference in FMD between hypertensive pregnancies and preeclampsia and therefore the groups were pooled. FMD was slightly but nonsignificantly higher in hypertensive group than in the reference group during pregnancy and practically equal postpartum.

The concentrations of the hsCRP, IL-6, and TNF-*α* are demonstrated in Table 3. All three markers of inflammation were elevated in hypertensive pregnancies compared to the postpartum situation of the same group. Also in normal pregnancies hsCRP, IL-6, and TNF-*α* concentrations were increased as compared to postpartum levels, but the differences were less pronounced and nonsignificant.

During normal pregnancy FMD% was associated negatively especially with brachial artery diameter at rest (*R* = −0.623, *P* < 0.001) and in lesser extent with GTN (*R* = −0.388, *P* = 0.028). Maternal weight near term was negatively associated with FMD% (*R* = −0.449, *P* = 0.041). HDL correlated to the dilation in nitro test. (*R* = 0.364, *P* = 0.041). Levels of hsCRP and IL-6 had a positive association, (*R* = 0.694, *P* < 0.001) and IL-6 correlated also with gestational age (*R* = 0.418, *P* = 0.021).

3 months after normal pregnancy baseline brachial artery diameter correlated negatively with FMD% (*R* = −0.487,  *P* = 0.016). Dilation in nitro test (endothelium independent vasodilation) correlated positively with blood pressure (*R* = 0.482, *P* = 0.017). We found no explaining factors for markers of inflammation postpartum.

In the group of hypertensive pregnancies brachial artery diameter at rest affected to GTN% (*R* = −0.555, *P* = 0.004) and hsCRP (*R* = −0.459, *P* = 0.021), whereas its association with FMD% was not significant (*R* = −0.177, *P* = 0.388). GTN% was also negatively associated with maternal age (*R* = −0.581, *P* = 0.002). Concentration of IL-6 was again associated with gestational age (*R* = 0.462, *P* = 0.017) and TNF-*α* (*R* = 0.424, *P* = 0.031). Baseline diameter was inversely associated with hsCRP (*R* = −0.459, *P* = 0.021).

Postpartum in the hypertensive pregnancies group FMD% correlated negatively to baseline diameter (*R* = −0.585, *P* = 0.017) and positively to TNF-*α* (*R* = 0.673, *P* = 0.017). Dilation in nitro test was negatively associated to baseline diameter (*R* = −0.671, *P* = 0.006). Concentration of hsCRP was associated with weight (*R* = 0.706, *P* = 0.010).

## 4. Discussion

The new finding of the present study was that brachial artery FMD was not attenuated in women with mild to moderate preeclampsia and hypertension in pregnancy or postpartum. In earlier studies endothelial dysfunction was present several weeks before clinical manifestation of preeclampsia and also several years after parturition in severe preeclampsia as compared to normal pregnancies [10]. To our knowledge there is only one previous study on the endothelial function in present preeclampsia or pregnancy-related hypertension [11].

The significantly lower birth weight (3597 versus 2907 grams) can be explained with slightly earlier delivery 40 versus 38 weeks in normal versus hypertensive pregnancies and also with placental dysfunction in late pregnancy in hypertensive pregnancies. Partly the difference was iatrogenic as the policy was to induce the labour at the onset of preeclampsia. However, the preeclampsia group of women had mild to moderate disease since in severe disease the protocol of this study would have been too challenging and therefore the differences between the birth weight and weeks at delivery were not clinically relevant.

Koopman et al. investigated microvascular endothelial function in women with intrauterine growth restriction (IUGR) with Laser Doppler fluxmetry using iontophoresis of acethylcholine (ACh) and sodium nitroprusside, and their finding was that endothelium-dependent (ACh-mediated) vasodilation was significantly increased in women with IUGR [12]. The investigators hypothesised that constant vasoconstriction in microcirculation of the IUGR mothers was the reason to improved endothelial dilation with ACh, indicating that endothelial dysfunction was behind the increased dilation. In the present study we used other methodology and probably the vascular bed also was different from that seen in IUGR, but this is the only study along with the present study showing an increase in endothelium dependent vasodilation not only in normal but also in complicated late pregnancies. Based on our findings we believe that the endothelial dysfunction that no doubt was present in hypertensive, preeclamptic, and also in IUGR pregnancies was not similar to endothelial dysfunction found in cardiovascular diseases and not necessarily even detectable noninvasively in conduit arteries in late pregnancy.

Endothelial dysfunction has been previously shown to be present in obese mothers (BMI > 30 kg/m^2^) in all three trimesters of pregnancy and four-month postpartum [13], and also in heavy smokers in the third trimester of pregnancy [14]. However, it is likely that the endothelial dysfunction in these cases is related to the metabolic phenotype of these women and not to the placental phenotype. On the other hand, traditional risk factors do not have an effect on endothelial function during pregnancy, since we have shown in a previous cohort study, that despite marked pregnancy-related hyperlipidemia, endothelial function was improved in healthy women and in normal pregnancies [4].

Our present work suggested that endothelial function in conduit arteries is similar in normal and in hypertensive pregnancies. In both groups endothelial function was better during pregnancy than postpartum, and in hypertensive pregnancies endothelial vasodilation after stimulus was even better than in normal pregnancies. The endothelial dysfunction in hypertensive pregnancies and in mild to moderate preeclampsia was not detectable in the brachial artery. It is possible that the increased blood flow in hypertensive pregnancies results in increased shear stress and thus even to greater increase in shear-stress mediated increased vasodilation. In a previous study, pulsed Doppler findings of maternal uterine artery, maternal ophthalmic artery, and brachial artery flow-mediated vasodilation varied among women with preeclampsia showing that there were vascular changes concomitantly or separately in uterus, ophthalmic vessels and conduit arteries [15]. In the microvascular bed, the endothelial dysfunction is easily detectable and the oedema caused by leaking endothelium could interfere with the measurements. Most probably the compromised nitric oxide production causing vasoconstriction is not the only mechanism leading to pregnancy-induced hypertension or preeclampsia, as we found increased FMD also in late hypertensive pregnancies.

We found that brachial artery diameter at rest had a negative correlation to FMD, because the small arteries dilate more than the larger ones. In normal pregnancies, near-term weight correlated also negatively to FMD. After hypertensive pregnancies TNF-*α* had a positive correlation to FMD but otherwise the correlations between proinflammatory cytokines and FMD were poor.

In this longitudinal study we found that even in normal pregnancies the third trimester concentrations of hsCRP, IL-6, and TNF-*α* were increased as compared to the postpartum values. The difference during pregnancy was not statistically significant between normal and hypertensive pregnancies though we noticed an increase in hsCRP and IL-6 concentrations in hypertensive pregnancies. This negative result may have occurred due to the type 2 error, meaning that sample size was too small to confirm the difference. From pregnancy to 3-month postpartum all the investigated markers of inflammation declined significantly in hypertensive women. The result verifies that pregnancy-induced hypertension and PE represent to some degree an inflammatory response to the pregnancy. However, the markers of inflammation were measured from systemic blood samples and thus the results do not necessarily represent the concentrations in various tissues as the cytokines are often released locally.

In conclusion, the present study showed that the endothelial response to a hypertensive pregnancy measured by FMD in the brachial artery was somewhat unexpected suggesting rather improvement than attenuation in endothelial function as compared to women with uncomplicated pregnancies. The present findings suggest that instead of endothelial dysfunction in conduit arteries other mechanisms related to systemic inflammation may play more important roles in the pathogenesis of gestational hypertension and mild-to-moderate preeclampsia.

## Figures and Tables

**Table 1:** Clinical characteristics of the 60 women examined during pregnancy and neonatal outcomes. Data is presented as means or number of cases (%), minimums, maximums, and standard deviations.

	Control, normal pregnancies (*N* = 32)	Hypertensive pregnancies (*N* = 16)	Preeclamptic pregnancies (*N* = 12)	*P* (Kruskall-Wallis test/Chi-square)	Pooled (*N* = 28)	*P* (Mann-Whitney *U*-test)
Age (years)	31 (20–39) ± 4.7	30 (21–41) ± 5.9	32 (25–40) ± 5.3	0.565	31 (21–41) ± 5.6	0.829
Gestational age at the examination day	34 (24–38) ± 3.26	36 (31–38) ± 1.72	33 (25–39) ± 4.53	0.027	35 (24–39) ± 3.53	0.187
Nulliparous (*n*/%)	18/56.3%	14/87.5%	5/41.7%	0.031	19/68%	0.356 (Chi-square)
BMI before pregnancy (kg/m^2^)	22.9 (18.7−28.6) ± 2.91	24.4 (19.3−33.8) ± 4.00	25.5 (19.7−32.5) ± 3.76	0.112	24.9 (19.3−33.8) ± 3.87	0.069
Gestational age at birth (weeks)	40 weeks (38−42) ± 10 days (1.4 weeks)	37 weeks (33−39) ± 6 days (0.9 weeks)	39 weeks (35−40) ± 11 days (1.5 weeks)	<0.001	38 (33−40) ± 11 days (1.6 weeks)	<0.001
Birth weight (grams)	3597 (2360−4700) ± 508	2754 (1775−3620) ± 533	3113 (1975−3960) ± 564	<0.001	2907 (1775−3960) ± 565	<0.001
Caesarean section (*N*/%)	5/15.6%	6/37.5%	3/25.0%	0.134	9/32.1%	0.131 (Chi-square)
Apgar at age of 1 minute/five minutes	9/9	9/9	8/9	0.276/0.831	8/9	0.150/0.370
Blood pressure	109/69 (91−125/57−81) ± 7.53/6.28	136/89 (118−149/81−99) ± 9.47/5.55	130/88 (107−146/64−99) ± 12.7/11.9	<0.001	134/89 (107−149/64−99) ± 11.0/8.70	<0.001

**Table:** (a) FMD during pregnancy and postpartum, data are expressed as means, (minimums, maximums) and standard deviations. Normal pregnancies compared to hypertensive pregnancies

	FMD (%)		*P*
	Pregnancy	Postpartum	
Control group (*N* = 32/24)	8.8 (−1.5–21) ± 5.1	7.9 (2.9–16) ± 3.5	*P* = 0.439
Hypertensive pregnancies (*N* = 26/16)	11 (−2.9–24) ± 6.3	8.0 (1.6–16) ± 4.2	*P* = 0.105
*P*	*P* = 0.194	*P* = 0.978	

**Table:** (b) FMD in normal, hypertensive end preeclamptic women during pregnancy and postpartum. The mean, minimums, maximums, and standard deviations

	Mean baseline diameter of the a. brachialis in pregnancy (mm)	Mean FMD during pregnancy %	Mean FMD in nitrotest during pregnancy	Mean baseline diameter of the a. brachialis postpartum (mm)	Mean FMD postpartum %	Mean FMD in nitro test postpartum
Normal pregnancy (*N* = 32/24)	0.33 (0.27–0.43) ± 0.04	8.8%	22%	0.30 (0.25–0.38) ± 0.03	7.9%	26%

Hypertensive pregnancy (*N* = 14/9)	0.35 (0.30–0.45) ± 0.04	10%	19%	0.32 (0.29–0.36) ± 0.03	8.5%	26%

Preeclamptic pregnancies (*N* = 12/7)	0.31 (0.24–0.34) ± 0.03	11%	21%	0.30 (0.25–0.34) ± 0.04	7.2%	28%

Difference btw the groups	HT versus PE *P* = 0.008	NS/HT versus PE *P* = 0.003	NS	NS	NS/NS	NS

pregnancy versus postpartum				Control *P* < 0.001, HT *P* = 0.001, PE NS	PE *P* = 0.04	Control *P* = 0.005 HT *P* < 0.001 PE *P* = 0.004

**Table:** (c) FMD during pregnancy and postpartum. Means, minimums, and maximums and SDs

		Pregnancy	Postpartum	*P*
Brachial artery diameter at rest (cm)	Control (*N* = 32/24)	0.33 (0.27–0.43) ± 0.04	0.30 (0.25–0.38) ± 0.03	<0.001*
	Hypertensive (*N* = 26/16)	0.33 (0.24–0.45) ± 0.04	0.31 (0.25–0.36) ±0.03	0.011*
	*P*	0.988	0.534	

Endothelium dependent vasodilation, FMD% (%)	Control (*N* = 32/24)	8.8 (−1.5–21) ± 5.1	7.9 (2.9–16) ± 3.5	0.439
	Hypertensive (*N* = 26/16)	11.0 (−2.9–24) ± 6.3	8.0 (1.6–16) ± 4.2	0.105
	*P*	0.194	0.978	

Endothelium independent vasodilation, NTG (%)	Control (*N* = 32/24)	22%	26%	0.005*
	Hypertensive (*N* = 26/16)	20%	28%	<0.001*
	*P*	0.221	0.421	

*: *P* value <0.05.

**Table 3:** Concentrations of hsCRP, IL-6, and TNF-*α* during pregnancy and postpartum. Medians (minimum-maximum).

		Pregnancy	Postpartum	*P*
hsCRP (mg/L)	Control *N* = 21/14	3.0 (0.54–13.4)	2.4 (0.22–7.0)	0.212
	Hypertensive *N* = 24/12	4.5 (0.89–38)	0.80 (0.27–5.9)	0.023*
	*P*	0.328	0.879	

IL-6 (pg/mL)	Control *N* = 21/14	1.8 (0.64–5.4)	1.3 (0.31–5.1)	0.118
	Hypertensive *N* = 24/12	2.1 (0.70–11)	1.2 (0.73–3.0)	0.006*
	*P*	0.137	0.879	

TNF-*α* (pg/mL)	Control *N* = 21/14	2.0 (1.2–4.1)	1.8 (1.1–4.0)	0.451
	Hypertensive *N* = 24/12	1.9 (0.88–3.2)	1.5 (0.71–2.1)	0.030*
	*P*	0.595	0.101	

*: *P* value <0.05.
